# Communication for behavioural impact in enhancing utilization of insecticide-treated bed nets among mothers of under-five children in rural North Sudan: an experimental study

**DOI:** 10.1186/s12936-016-1551-8

**Published:** 2016-10-18

**Authors:** Yousif Mohammed Elmosaad, Magda Elhadi, Asif Khan, Elfatih Mohamed Malik, Ilias Mahmud

**Affiliations:** 1 Faculty of Medicine, Gezira University, Wadmadni, Sudan; 2 College of Public Health & Health Informatics, Qassim University, Bukayriah, Qassim, Saudi Arabia; 3 Federal Ministry of Health, Khartoum, Sudan; 4 James P Grant School of Public Health, BRAC University, Dhaka, Bangladesh

**Keywords:** Malaria, Communication for behavioural impact, Mothers of under-five children, Long-lasting insecticidal nets, North Sudan and utilization

## Abstract

**Background:**

Malaria is the leading cause of morbidity and mortality in Sudan. The entire population is at risk of contracting malaria to different levels. This study aimed to assess the effectiveness of communication for behavioural impact (COMBI) strategy in enhancing the utilization of long-lasting insecticidal nets (LLINs) among mothers of under-five children in rural areas.

**Methods:**

A randomized community trial was conducted in rural area of Kosti locality, White Nile State, Sudan, among mothers of under-five children, from January 2013 to February 2014. A total of 761 mothers from 12 villages were randomly selected, 412 mothers from intervention villages and 349 were from comparison villages.

**Results:**

The knowledge of mothers, in intervention villages, about malaria vector, personal protective measures (PPM) against malaria, and efficacy of LLINs was significantly increased from 86.9 to 97.3 %; 45.9 to 92 % and 77.7 to 96.1 % respectively. Knowledge about usefulness of PPM, types of mosquito nets and efficacy of LLINs was significantly higher in intervention villages compared to comparison villages (p < 0.05), (η^2^ = 0.64). Mothers in intervention villages increasingly perceived, post-intervention, that malaria was a serious disease (99.3 %), a preventable disease (98.8 %) and also LLINs as an effective intervention in malaria prevention (92.2 %). This resulted in an increase in the utilization rate of LLINs from 19.2 to 82.8 % in intervention villages compared to comparison villages (p < 0.05) [OR = 4.6, 95 %, CI = (3.72–5.72)], (η^2^ = 0.64). The average of mothers’ knowledge about malaria was increased by 64 % (η^2^ = 0.64), the use of LLINs was increased by 79 % (η^2^ = 0.79) and a positive attitude towards malaria was 2.25 times higher in intervention villages than among mothers in the comparison villages.

**Conclusions:**

These results established the usefulness of COMBI strategy for increasing awareness about malaria, developing a positive perception towards malaria prevention and, increasing the utilization of LLINs.

**Electronic supplementary material:**

The online version of this article (doi:10.1186/s12936-016-1551-8) contains supplementary material, which is available to authorized users.

## Background

In Northern Sudan, malaria is a major public health problem [1]. It is a leading cause of morbidity and mortality. Nearly 75 % of the total population is at risk of malaria, and malaria endemicity varies from hypo-endemic in the north to hyper-endemic in some areas in the south [2]. In Northern Sudan, malaria incidence is high among pregnant women and children under five years of age. Around 21 % of outpatient consultations, 30 % of inpatient admissions and 16 % of hospital deaths are attributed to malaria [3]. Studies of individual hospitals in Northern Sudan have found case fatality rates for malaria between 5 and 12 %, with children under 3 years of age four times more likely to die than the older ones [4]. The overall parasite prevalence has doubled in 2012 compared to 2009 (increased from 1.8 % in 2009 to 3.3 % in 2012) [4]. Over 90 % of malaria cases are caused by *Plasmodium falciparum* and *Plasmodium vivax* is less common. *Anopheles arabiensis* is the principal vector with focal contributions by *Anopheles gambiae* and *Anopheles funestus* [5].

Various interventions have been used to prevent malaria. The use of mosquito net is one of them. This has been improved by using insecticide-treated nets (ITNs), which have been found to be more effective [6]. Based on series of field studies of the effectiveness of ITNs on malaria morbidity and mortality in sub-Saharan Africa, promotion of use of ITNs has emerged as a key intervention for malaria control. This large-scale use of ITNs reduced clinical malaria episodes by 48 to 50 % [7–10].

Since 2002, following the establishment of The Global Fund to Fight AIDS, Tuberculosis and Malaria (GFATM) considerable investment has been made to scale up malaria control interventions in Africa which has led to substantial increase in the coverage of key malaria interventions, namely ITNs, especially the long-lasting insecticidal nets (LLINs) [11].

In Sudan, results from the malaria indicator survey in 2012 reflected that significant effort of the Federal Ministry of Health in scaling up malaria prevention and control interventions increased ITN ownership and use, as well as knowledge of malaria [4]. Although, more than 20 million ITNs were distributed between 2005 and 2009 that enabled 68 % of the households living in malaria-endemic areas to own at least one ITN [12], it was far less than the target of 80 %, and only 20 % of the general population in that area were sleeping under ITNs. The number who actually slept under ITNs at night was even less [5]. With this situation, important challenges remained in efforts to control malaria [12].

To explain the ineffective utilization of ITNs particularly among rural communities, it had been found that there was a limited data on knowledge, attitudes and practice of households about ITNs. Hence, rural Kosti locality was selected for this study to assess the effectiveness of the “Communication for Behavioural Impact” (COMBI) strategy for enhancing the knowledge, attitudes, and practices in malaria prevention among the mothers of under-five children and hence increasing the utilization rate of ITNs.

## Methods

### Study design and sites

A randomized community trial was conducted in rural areas of the Kosti Locality, White Nile State, Sudan from January 2013 to February 2014 to assess the effectiveness of the COMBI strategy in enhancing utilization of LLINs among the mothers of under-five children.

White Nile State is one of the Sudan’s 18 states. It lies in the South of the country, and bordered by Khartoum state from the north, South Sudan from the South, South Kordfan state from west, Blue Nile State from south east and from the east Senar, Gazera. This location makes the state susceptible to malaria transmission throughout the year as this area is an agricultural one. Primary malaria control and prevention activities in White Nile state are undertaken by the State Ministry of Health, the National Malaria Control Programme (NMCP) together with non-governmental organizations (NGOs). Kosti locality is one of White Nile state localities and it covers a total area of 1220 sq km with a total population of 179,800.

### Study population and sampling

The study population comprised of mothers of under-five children who were living in rural areas (villages) of Kosti locality. Mothers having plans of leaving the area during the course of the study were excluded. The study sample was determined by using the following formula: n = Z2PQ/D2 [13] and was found to be 761 mothers of under-five children, where Z is the corresponding value to 95 % confidence limits = 1.96, P = utilization rate of LLINs (0.45), Q = 1 − P = (0.55), D = desired margin of error (absolute precision) = 0.05, and Deff = 2.

Out of a total of 163 villages in Kosti locality, 12 villages were selected according to the NMCP criteria for LLINs distribution and were used for this study. These criteria were villages near to White Nile river bank which were surrounded by farms and having a mosquito density all year round- four mosquitoes per room, 3.8 larvae per dip. Randomly, six villages located in North West region of the Kosti locality were assigned to the intervention group and six villages located in South East region of the Kosti locality were assigned to the comparison group. Both groups were assessed twice during the study period: pre-intervention and post-intervention. The calculated total sample size (761) was divided into comparison and intervention group proportionate to population size and hence 412 (54.1 %) mothers from a total of 1720 mothers in intervention villages and 349 (45.9 %) from a total of 1460 mothers in comparison villages were selected for the study. Systematic random sampling was used-every fourth mother was selected from the list of mothers prepared by the NMCP prior to distribution of LLINs.

### Phases of the study

The study included three phases, namely pre-intervention phase, intervention phase and post-intervention phase.

During pre-intervention phase, baseline data was collected in the last week of January 2013 from randomly selected mothers of under-five children using a structured questionnaire that included socio-demographic characteristics of households, KAP of mothers related to malaria prevention and villages’ environment, sleeping behaviour of mothers and general conditions and utilization of LLINs (see Additional file 1). In addition, baseline malaria parasitological data were collected during the malaria transmission season in the 1st week of February 2013 from both intervention and comparison groups. One child of less than 5 years of age was selected randomly from each household from where the mothers were included in the study sample. Two drops of blood were taken from each selected child, one drop was wiped off from the finger using a swap dipped into methylated sprit and the other drop was used for testing in the field using rapid diagnostic test to detect *P. falciparum*. Before baseline data collection, during the first 10 days of January 2013, a total numbers of 13,650 LLINs were distributed in both intervention and comparison villages. Different household received different numbers of LLINs depending on its population size- one net for two members.

#### Intervention phase

During the first week of January 2013, local committee and health promotion teams in intervention villages were formed. The locality committee members included staff from the public health unit at locality level: administrative staff, public health officers, health education specialists, entomologists and community leaders. The committee was responsible for providing training, mobilizing the village health promotion teams including monitoring and support for the COMBI activities in intervention villages. The health promotion teams consisted of women group leaders, female primary school teachers and government community health volunteers.

During the following week, a week-long training workshop was held for the six health promotion teams in the intervention villages. The workshop sessions were participatory in nature to increase knowledge of health promotion team members regarding LLINs. These workshops were facilitated by the trainers from the malaria control programme of the federal ministry of health, staff from the malaria control programme of the state Ministry of Health, and health education specialist from the faculty of public and environmental health, University of Khartum. The participants from the intervention villages returned to their villages to carry out the COMBI activities. Each health promotion team member at the village level was responsible for ten mothers of under-five children. Each health promotion team was supervised by a co-ordinator—a primary school teacher.

### Intervention COMBI activities

COMBI activities were carried out for a period of 10 months between February and December 2013. COMBI activities were implemented from different points of view as summarized below:

Personal selling was done through village health promotion teams. For every ten mothers of under-five children in intervention villages, a volunteer made house to house visits with the frequency being once in every month for a period of 10 months to create awareness about the importance, use and maintenance of LLINs.

Focus group discussions were conducted by the locality committee and health promotion teams with all sampled mothers of under-five children (412) in the intervention villages. The FGDs were about malaria and its prevention modalities including the importance, use and maintenance of LLINs. Each FGD consisted of 8–10 mothers and was facilitated by the head of the health promotion team and one malaria prevention specialists from the locality committee. FGDs were repeated for reinforcement after six months of the initial FGD.

Sustained massive advertising: Distribution of health education materials, including posters, pamphlets and books, to mothers of under-five children in intervention villages in women public places, schools, primary health care centres and households having mothers of under-five children.

Health education sessions were conducted monthly through lectures, group discussions and workshops by health promotion teams regularly and by local committee occasionally to mothers of under-five children and school children in the intervention villages. The health education sessions were conducted following the manual developed by the federal ministry of health.

Community mobilization was done by health promotion teams through monthly mothers’ group meetings and home visits.

Administrative mobilization was done monthly by the locality committee with each health promotion team in intervention villages with a purpose of mobilizing the teams through meetings and discussions.

At the post-intervention phase, in January 2014, the same questionnaire which had been used for baseline data collection was used for data collection from intervention and comparison groups. While the end-line malaria parasitological data were collected during the malaria transmission season in the first week of February 2014 from both intervention and comparison groups. Like baseline data, one child of less than five years of age was selected randomly from each household from where the mothers were included in the study sample. The same group of data collectors, who did baseline data collection, collected end-line data. Both baseline and end-line survey was conducted during malaria season and in the same months, last week of January, in the year 2013 and 2014, respectively. In order to avoid bias in response to the questionnaire, data collectors were not involved in COMBI activities. Data collectors received training to avoid leading questions. A structured questionnaire was used and data collectors just read out the questions and recorded respondents’ response without providing any further clue.

### Data analysis

Depending on the nature of the variables, descriptive statistics were used to tabulate and describe the data (frequency distribution, percentages, means and standard deviations), and inferential statistics (Chi Square and Fisher’s exact tests) was used to examine association between categorical variables. Odds Ratio (OR) and Eta Square (η^2^) were calculated to determine effect size of independent variables on dependent variables.

Since the data was normally distributed Z-value lies between (−1.96 and +1.96), one way ANOVA and independent sample *T* test were done to explain the differences in COMBI outcome and KAP scores by socio-demographic characteristics. Linear regression analysis was used to predict the effect of mothers’ knowledge on their attitude and practices of preventive measures against malaria. Multiple logistic regression analysis was performed to see the effect of the COMBI.

The data was analysed using the Statistical Software Package for Social Sciences (SPSS) version 18. For close-ended questions of the questionnaire, the replies of equivalent meaning were pooled into different categories during analysis and percentages were computed for different options. The tests were carried out at 95 % confidence interval, p values less than 0.05 were considered significant, and OR with 95 % confidence interval was used to assess the presence and degree of association between dependent and independent variables. Multiple logistic regression was performed using the Stata/SE 13.1.

### Ethical considerations

Data collectors were trained on ethical issues- obtaining verbal informed consent, respecting mothers’ decision of not participating in the study, ensuring participants’ privacy and confidentiality of information provided. Verbal informed consent was obtained from each participant before enrolling them in the study. At the end of each day data collection, all hard copies of completed questionnaires were submitted to the health promotion team co-ordinators in each village and the researcher for daily review for consistency and quality of data, and to safeguard confidentiality of the information provided. All malaria positive cases detected during data collection were given, by a qualified nurse, an appropriate age-weight specific treatment course as per the national treatment guidelines.

## Results

### Socio-demographic characteristics of the respondents

Of the 761 participants in the study, in both intervention and comparison villages, almost half had only primary education (49 and 45.6 % respectively), while over a quarter (28.4 %) did not have any formal schooling. Majority of the respondents’ husbands were on irregular free job both in intervention (63.6 %) and comparison villages (62.2 %) and the mean household monthly income was 68.8 and 77.1 USD, respectively in intervention and comparison groups. In intervention and comparison group, respectively, the mean age of the mothers were 29.3 and 30.3 years; average family size was 5.3 and 5.6; and average number of under-five children was 1.6 and 1.7. No statistically significant difference was found between intervention and comparison group on socio-demographic variables (see Table 1).

**Table 1:** Socio-demographic characteristics of the respondents

Household characteristics	Intervention villages (n = 412)	Comparison villages (n = 349)	p value
Husband’s occupation			
Government employee	35 (8.5)	22 (6.3)	0.125
Business	30 (7.3)	34 (9.7)	
Farmer	85 (20.6)	76 (21.8)	
Free job	262 (63.6)	217 (62.2)	
Education level			
Informal education	117 (28.4)	99 (28.4)	0.529
Primary education	202 (49.0)	159 (45.6)	
Intermediate	23 (5.6)	24 (6.9)	
Secondary	60 (14.6)	53 (15.2)	
University	10 (2.4)	14 (4.0)	
Monthly income (USD)	68.8 ± 39.6	77.1 ± 41.2	0.074
Age of mothers	29.3 ± 6.7	30.3 ± 7.9	0.148
Family size	5.3 ± 2.1	5.6 ± 2.2	0.071
Number of children <5 years	1.6 ± 0.7	1.7 ± 0.7	0.416

Figures in parenthesis indicate percentage

Plus-minus values are means ± standard deviations (SD)

### Knowledge of mothers about malaria

Majority of mothers were found to have correct knowledge about malaria vector both before and after intervention. However, there was a significant increase in knowledge about malaria vector among mothers in intervention villages from 86.9 % before intervention to 97.3 % after intervention and hence the knowledge level increased by 11 % (Eta square η^2^ = 0.11). No such significant increase was observed in comparison group of mothers.

In intervention villages, 82.5, 43.9 and 45.6 % mothers knew fever, joint pains, vomiting and diarrhoea respectively as the symptom of malaria before intervention, while after intervention the respective knowledge of mothers significantly improved to 98.3, 85.7 and 86.7, respectively (p < 0.05). 25 % mothers cited one of the household members had been infected by malaria before intervention that significantly reduced to 18 % after intervention (p < 0.05).

Also, the results revealed that 34 % mothers cited not using LLINs continuously as the major reason for getting malaria infection which significantly increased to 47.3 %, after intervention, while 9.7 % mothers cited never using LLINs as the second reason to get malaria infection which significantly increased to 32.4 %, after intervention (p < 0.05). This indicates that level of knowledge significantly increased by 22 % after intervention (η^2^ = 0.22). With regard to knowledge about personal protective measures against malaria, 85 % of mothers stated that they used LLINs as personal protective measure, which significantly increased to 92.1 % after intervention (p < 0.05).

Related to recognizing the types of mosquito nets, most of mothers in intervention villages (84.3 %) recognized all types of mosquito nets than the mothers from comparison villages (11.2 %) (p < 0.05). This also indicates that level of knowledge increased by 64 % following intervention (η^2^ = 0.64). Also, 47 % mothers recognized all types of ITNs before intervention which significantly increased to 56.8 % after intervention (Table 2).

**Table 2:** Pre and post-intervention knowledge of mothers about malaria

Variables-knowledge about malaria	Response	Intervention villages (n = 412)		χ^2^	p value	Comparison villages (n = 349)		χ^2^	p value
		Pre (%)	Post (%)			Pre (%)	Post (%)		
Malaria vector	Mosquitoes	86.9	97.3	45.9	p < 0.001	88.5	89.1	13.8	p > 0.127
Malaria transmissions	Mosquito biting	78.4	95.9	59.9	p < 0.001	81.1	85.7	6.57	p > 0.127
Symptoms of malaria	Fever	82.5	98.3	28.1	p < 0.001	79.9	79.1	0.390	p > 0.940
	Joint pains	43.9	85.7			41.8	45.8		
	Vomiting and diarrhoea	45.6	86.7			43.6	44.4		
	All of the above	11.2	75.7			12.3	15.2		
Household malaria prevalence	Yes	25	18	101.6	p < 0.001	24.6	23.8	0.284	p > 0.0867
Perceived reasons why household members got malaria infection	Never used ITNs	9.7	32.4	28.2	p < 0.001	19.8	25.3	1.54	p > 0.670
	Irregular use of ITNs	34	47.3			24.4	21.7		
Personal protective measures	Repellents	45.9	92	9.0	p < 0.05	46.7	52.4	0.360	p > 0.985
	LLINs	85	92.1	20.7	p < 0.001	85.5	85.8	0.17	p > 0.991
Recognizing the types of nets	Ordinary	29.8	4.1	340.8	p < 0.001	30.6	31	0.33	p > 0.084
	Impregnated	55.8	11.6			56.5	57.8		
	Both responses	14.4	84.3			12.9	11.2		
Types of impregnation	LLINs	21	22.9	8.17	p < 0.017	19.8	20.1	0.245	p > 0.885
	ITNs	32	20.3			30.8	33		
	Both responses	47	56.8			49.4	46.9		

### Attitudes of mothers towards malaria

Regarding malaria as a preventable disease, Table 3 depicts that 80.6 % of mothers perceived malaria as a preventable disease which significantly increased to 99.3 % after intervention, (p < 0.05), (η^2^ = 0.30). 75.2 % mothers recognized malaria as a serious disease before intervention, which significantly increased to 98.8 % after intervention (p < 0.05). In intervention villages, the results showed that the negative attitude of mothers about LLINs with large holes (damaged LLIN) protecting from malaria significantly reduced from 32.5 to 12.4 %, after intervention (p < 0.05) (η^2^ = 0.24).

**Table 3:** Attitudes of mothers towards malaria

Attitudes of mothers towards malaria	Intervention villages (n = 412)		χ^2^	p value	Comparison villages (n = 349)		χ^2^	p value
	Pre (%)	Post (%)			Pre (%)	Post (%)		
Malaria is a preventable disease	80.6	99.3	79.55	p < 0.001	81.7	83.1	0.169	p > 0.91
Malaria is a serious disease	75.2	98.8	101.5	p < 0.001	75.1	79.7	2.12	p > 0.34
Damaged LLINs also protect from malaria	32.5	12.4	48.01	p < 0.001	33.5	29.8	1.12	p < 0.16
LLINs can be an effective means against mosquito biting	55.8	92.2	136.2	p < 0.001	54.2	59.3	1.89	p < 0.097
Mothers encourage family members to use LLINs	52.7	93.7	176.6	p < 0.001	52.7	56.2	0.832	p < 0.362
LLINs is one of the preventive measures against malaria	77.7	96.1	62.36	p < 0.001	87.8	79.9	0.362	p < 0.830

56.8 % of the mothers had a positive perception about effectiveness of LLINs in preventing against mosquito bites that significantly increased to 92.2 % after the intervention (p < 0.05) (η^2^ = 0.41). Also, 52.7 % mothers encouraged their family members to use LLINs before intervention which significantly increased to 93.7 % after intervention (p < 0.05). Likewise, 77.7 % mothers had positive perception about effectiveness of LLINs in prevention of malaria that significantly increased to 96.1 % after intervention (p < 0.05).

While in comparison villages, 54.2 % mothers’ perception about effectiveness of LLINs as preventive measure against mosquito biting at the time before intervention slightly increased to 59.3 % at the time after intervention (p > 0.05). 52.6 % mothers encouraged their family members to use LLINs at the time before intervention, which slightly increased to 56.2 % at the time of after intervention (p > 0.05). On the other hand, in the perception of mothers about effectiveness of LLINs in prevention of malaria, there was no significant change (p > 0.05).

### Utilization of long-lasting insecticidal nets

**Fig. 1:**
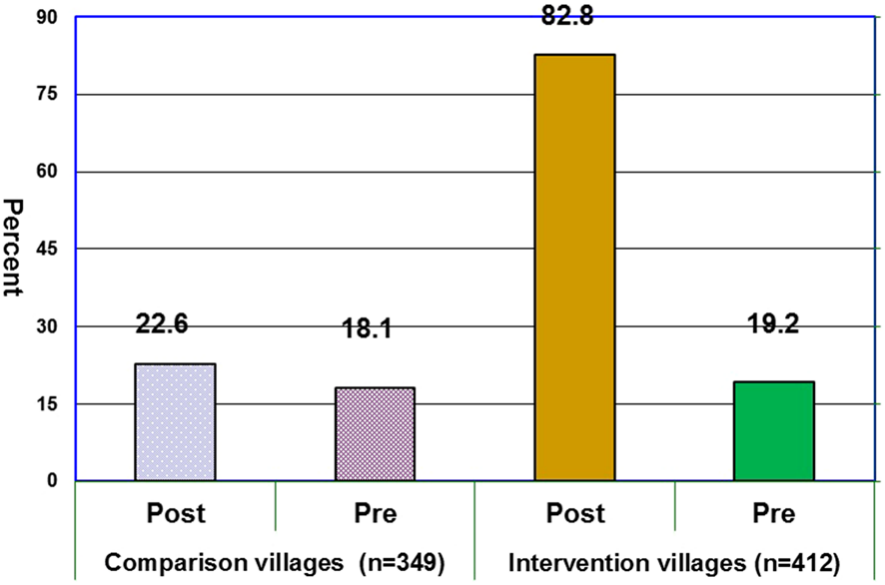
Utilization of LLINs amongst mothers of under-five children

Figure 1 presents data on LLINs utilization amongst mothers of under-five children in households that owned nets. The questions focused on daily use of LLINs. In intervention villages, before COMBI intervention 19.2 % mothers reported that they had slept under a bed net which significantly increased to 82.2 % after intervention, (p < 0.05), [OR = 4.6, CI = (3.72–5.72)]. This indicates 64 % improvement in LLIN utilization following COMBI activities (η^2^ = 0.64). In comparison villages, the percentage of households using LLINs appeared to be slightly increased from 18.1 to 22.6 % at the time before intervention and after intervention, respectively (p > 0.05).

### Variables associated with LLINs utilization

The results presented in Table 4 revealed that the LLINs utilization was more in households who have sufficient knowledge about malaria (3.4 times) [OR = 3.4, 95 % CI (1.82–6.34)], households who had members with formal education (3.6 times) [OR = 3.6, 95 % CI (2.40–5.40)], Government employees (3.7 times) [OR = 3.7, 95 % CI (0.87–15.8)] and also in those who had less than three children (3.5 times) [OR = 3.5, 95 % CI (0.89–13.80)]. However, income and family size did not affect LLINs utilization (p > 0.05).

**Table 4:** Variables associated with LLINs utilization in intervention villages

Variable (n = 412)		LLINs utilization—regular users				χ^2^	OR (95 % CI)	p value
		Yes		No				
Knowledge status	Don’t have sufficient knowledge	4	1.0	5	1.2	9.47	3.4 (1.82–6.34)	0.010
	Have sufficient knowledge	337	81.8	66	16.0			
Educational level	Informal education	77	18.7	42	10.2	38.3	3.6 (2.4–5.4)	0.001
	Formal education	264	64.1	29	7.0			
Occupation status	Government employee	33	8.0	2	0.5	3.56	3.7 (0.87–15.8)	0.039
	Private employee	308	74.8	69	16.7			
Monthly income	Below mean (USD 72.5)	201	52.8	42	11.0	0.145	1.1 (0.65–1.90)	0.401
	Above mean	112	29.4	26	6.8			
Family size	Below mean (5.35)	132	32.2	31	7.6	2.04	0.82 (0.49–1.38)	0.360
	Above mean	207	50.5	40	9.8			
Number of children <5 years	1–2	303	73.9	69	16.8	4.25	3.5 (0.89–13.8)	0.024
	>3	36	8.8	2	0.5			

Maximum score for the ‘knowledge’ variable was 38 (one point for each right answer). A knowledge score of 20 or above was considered as sufficient knowledge

Table 5 demonstrates that after controlling for demographic variables there is a significant association between being in the intervention or in the comparison groups and the utilization of LLINs following 10 months of COMBI activities. Being in the intervention group was associated with a 0.59 points increase in the log of odds of using LLINs in comparison to the comparison group. This table also presents that there is a significant association between education and LLINs utilization. Formal education was associated with a 0.16 point increase in the log of odds of using LLINs in comparison to informal education following COMBI. No significant association was found between the LLINs utilization and other socio-demographic variables- occupation, household monthly income, household population and number of children under 5 years in the household. This model could explain 37 % of the total variation (adjusted R squared 0.37).

**Table 5:** Multivariate analysis of utilization of LLINs in intervention and comparison villages following COMBI

LLIN Usage	Coef.	Std. Err.	t	p > t	95 % Confidence interval	
Groups						
Comparison	0.00					
Intervention	0.59	0.03	19.49	0.00	0.53	0.65
Education						
Informal	0.00					
Formal	0.16	0.03	4.69	0.00	0.09	0.23
Occupation						
Government employee	0.00					
Business	0.01	0.07	0.14	0.89	−0.13	0.15
Farmer	−0.01	0.06	−0.15	0.88	−0.14	0.12
Free job	0.01	0.06	0.25	0.80	−0.10	0.13
Household monthly income	0.00	0.00	0.06	0.95	0.00	0.00
Household population	−0.01	0.01	−1.01	0.31	−0.02	0.01
Number of children <5 years	0.04	0.02	1.8	0.07	0.00	0.09
Constant	0.08	0.08	0.93	0.35	−0.09	0.24

Regarding overall knowledge about malaria among mothers in intervention villages there was improvement in knowledge from 3.8 to 34.2 % (p < 0.05) (η^2^ = 0.64). This signifies that level of knowledge increased by 64 % in intervention villages. However in comparison villages, there was no significant improvement (p > 0.05).

With respect to overall attitude about malaria among mothers in intervention villages 75.2 % of mothers had a positive attitude towards malaria, which significantly increased to 99.3 % after the intervention (p < 0.05). However 24.8 % of them had a negative attitude towards malaria, which significantly reduced to mere 0.7 % after the intervention. Mothers after the intervention developed a positive attitude towards malaria more than two times higher than mothers in the comparison villages (OR = 2.25). However, there was no significant change in overall malaria KAP score in the comparison villages (p > 0.05).

About the overall score of practice of malaria prevention in intervention villages, 0.8 % of mothers of under-five children, had good preventive practices against malaria that significantly increased to 33.6 % after the intervention (p < 0.05) (η^2^ = 0.79). While 32.4 % of them had poor preventive practices, that significantly reduced to zero after intervention (p < 0.05) (Table 6).

**Table 6:** Pre- and post-intervention levels of overall knowledge, attitude and practice of mothers about malaria

KAP	Variables	Intervention villages (N = 412)		Df	χ^2^	η^2^	p value
		Pre (%)	Post (%)				
Overall knowledge about malaria	Good knowledge	3.8	34.2	2	354.7	0.64	p < 0.001
Practice level of prevention measures	Good practice	0.8	33.6	2	52.3	0.79	p < 0.001
Attitude level of mothers*	Negative attitude	24.8	0.7	1	106.97	0. 36	p < 0.001
	Positive attitude	75.2	99.3				

Poor knowledge (1–12), Moderate knowledge (13–24) and Good knowledge (>25). Maximum score for the ‘knowledge’ variable was 38 (one point for each right answer)

An overall score of practice of malaria prevention was calculated by adding up the score for each of all thirteen questions and the maximum total score was 18 [Poor practice (1–6), Fair practice (7–12) and Good practice (13–18)]

To assess the attitude of mothers of under-five children towards malaria. The response to the questions was in the form of Yes or No and a positive attitude with a response “yes” was given two points and a negative attitude with a response “No” was not given any point

There was no significant change in level of knowledge, practice and attitude among mothers of under-five children living in comparison villages at the time of post-intervention (p > 0.05)

* 95 % Confidence interval: [OR = 2.25, CI (2.05–2.46)]

### Prevalence of malaria parasites among children under five years of age using rapid diagnostic test

Table 7 shows that in intervention villages prevalence of malaria among children reduced from 4.7 % before intervention to 2.3 % after intervention (p < 0.001). No significant change was seen among the comparison villages. Infection was higher among children in comparison villages (4.8 %) compared to children in intervention villages (2.3 %). It is an indication that COMBI was found to have significant influence on reduction of malaria prevalence [OR = 1.4 CI (1.05 − 1.9)].

**Table 7:** Malaria parasite prevalence using RDT among children under 5 years of age who were tested before and after intervention

Tests	n (%) positive for intervention villages^a^	n (%) positive for comparison villages^b^	χ^2^	OR (95 % CI)	p value
Pre-test	19 (4.7)	17 (4.9)	0.27	1.06 (0.54–2.07)	0.866
Post-test	9 (2.3)	16 (4.8)	3.52	1.4 (1.05–1.9)	0.047

^a^n = 398; 3.4 % children refused blood test

^b^n = 334; 4.2 % children refused blood test

## Discussion

Malaria is the leading cause of morbidity and mortality in Sudan [14]. The entire population of Sudan is at risk of contracting malaria, especially people living in rural areas. Risk is more in villages located near river banks—due to an increased mosquito density. The strategic approaches adopted by the NMCP in collaboration with the Roll Back Malaria (RBM) initiative, are vector control through ITNs, and supporting communications for behavioural change initiatives [15].

This study established the effectiveness of the Communication for Behavioural Impact (COMBI) strategy in mothers of under-five children. Intervention and comparison villages were randomly selected from two different regions (North West Region and South West Region) of the Kosti locality to minimize ‘contamination’ between intervention and comparison. Yet, this might also introduce bias due to ecological and demographical differences between the villages, which might influence the use of LLIN. However, both regions were ecologically similar and were selected following the NMCP criteria. Demographic analyses also suggest that study participants in intervention and comparison groups were not significantly different. The study findings indicated that following COMBI intervention utilization of LLINs increased. The results showed that at the baseline majority of the mothers had the knowledge of malaria vector in both intervention and comparison villages. Similar results, a higher proportion of mothers identified mosquitoes to be the sole cause of malaria, were found in Kinango, Uganda [15] and also in Iran [16].

Significant improvement was observed, following intervention, in mothers’ knowledge about signs and symptoms of malaria (fever, joint pains, vomiting and diarrhoea) in intervention villages. This is similar to the findings of a study done in Mali by Rhee et al. [17] and by Elsheikh [18] in Sudan.

Malaria prevalence was measured by asking about malaria infection in the previous two weeks of each survey. The results showed that a quarter of the mothers cited one of the household members had been infected by malaria before intervention that significantly reduced following intervention (Table 2). This is similar to the findings by Gabbad et al. in Sudan [19], Noor et al. in Central Somalia [20], as well as in southeast Nigeria by Igwe et al. [21].

In this study, among the mothers who cited the reasons for getting malaria, over a third of the mothers felt that not using LLINs continuously was a major reason followed by a one tenth of the mothers who mentioned never using LLINs as the second reason. Mothers’ knowledge in this regard significantly increased after intervention (p < 0.05; η^2^ = 0.22). This also indicates that majority of mothers believed that LLINs were effective in controlling malaria by avoiding and killing mosquitoes.

With regards to knowledge about LLIN as a personal protective measure (PPM), at baseline 85 % of mothers perceived LLINs as a PPM against malaria, which significantly increased to 92.1 % after intervention. Similar results were reported in Ethiopia [22] and in Nigeria [23], which found that LLIN use increased when individuals received health promotional activities and health education, respectively.

Pertaining to the mothers’ knowledge related to usefulness of PPM, types of mosquito nets and efficacy of LLINs, it was found that these significantly increased following intervention in intervention groups. In contrast, in the comparison villages no significant increase was observed regarding knowledge related to usefulness of PPM. Similar results have been reported by Lin et al. [24], showing an increased use of ITN following health promotional activities about malaria preventive measures.

Regarding the preventability of malaria, the results showed that at baseline 80.6 % of mothers perceived malaria as a preventable disease, which significantly increased to 99.3 % after intervention. Similar results were mentioned by Nwana in Cameroon [25].

Concerning the attitudes of mothers, towards malaria as a serious disease, 75.2 % of mothers acknowledged malaria as a serious disease before intervention, which significantly increased to 98.8 % after intervention. This is higher than that found in a study done in Nigeria which demonstrated that 55 % mothers viewed malaria as a very serious illness [26]. The results also showed that the negative attitude of mothers about ability of LLINs with damaged, large holes protecting from malaria significantly reduced from 32.5 to 12.4 %. This is in agreement with the findings of the study conducted by Irish in Benin in 2008 which showed there was an association between the proportion of mosquito bites and size and number of holes in the ITNs [27].

At baseline, over half of the mothers in the intervention villages had a positive perception about the effectiveness of LLINs in prevention against mosquito bites, which had significantly increased to 92.2 % after the intervention. This was higher than the studies conducted by Ndwiga et al. (75 %) [28], Ruberto et al. (56 %) [29], Kimbi et al. (57 %) [30] and by Appiah-Darkwah et al. (63.1 %) [31]. Nwana also found a higher percentage than other studies mentioned above where it was shown that 76.26 % individuals perceived some advantages for utilizing ITNs like preventing malaria, killing mosquitoes and avoiding nuisance by mosquito bites and also for a better sleep [25].

Also in this study, in the intervention villages over half (52.7 %) of the mothers encouraged their family members to use LLINs before intervention, which significantly increased to 93.7 % after intervention (p < 0.05). This was probably because, after educational intervention, the mothers recognized LLINs as an effective tool in prevention of malaria. Besides, 77.7 % mothers had a positive perception about effectiveness of LLINs in prevention of malaria that significantly increased to 96.1 % after intervention (p < 0.05). While 7.8 % of them had a negative perception, that significantly reduced to 0.7 % after intervention. The positive perception in this study was higher than the studies conducted by Soleimani-Ahmadi et al. (60.8 %) [16] and Kimbi et al. (99 %) [30].

The results showed that before intervention, only about one fifth of the mothers reported that they had slept under a bed net which significantly increased to 82.2 % after intervention, (p < 0.05), [OR = 4.6, CI = (3.72–5.72)] (η^2^ = 0.64). Similar results have been obtained in some other African countries and also in Asian countries by Lin et al. [24], Rhee et al. in Mali [17] and Elsheikh in Sudan [18], who used health education as intervention. Also a slightly lower result was reported by Berie et al. in Ethiopia, who found that the utilization of ITNs was 76.8 % [32], and a higher result was reported by Amoran, in Nigeria who found that the utilization of ITNs increased after health promotion message from 50.8 to 87.4 % (p < 0.001) [33], and also by Adebayo et al. in Nigeria who found that the utilization of ITNs was 83.5 % [34].

Also the effectiveness of health education in increasing the utilization of ITNs was seen in a controlled trial study in Piron, Mali with a 40 % increase in the level of utilization of ITNs in the village at the end of the interventional study [17]. Likewise post-intervention use of LLINs among the respondents that owned LLINs in this study (82.2 %) was higher than the ITNs usage of 52.1 % reported by Deressa et al. [35].

Related to the associated factors with LLINs utilization, the results revealed that the LLINs utilization was more in households who have sufficient knowledge about malaria (3.4 times) [OR = 3.4, 95 % CI (1.82−6.34)]. This is because if people knew about malaria infection and the prevention methods earlier they used LLIN as a preferred method to protect themselves from malaria infection. In this study, mothers having formal education were found to be 3.6 times more likely to utilize LLINs than those not having formal education [OR = 3.6, 95 % CI (2.40−5.40)]. This is similar to a study conducted in northwest Ethiopia by Berie et al. [32]. However, income and family size did not affect directly on LLIN utilization (p > 0.05). This is in contrast to a study conducted in Bahir Dar city, Ethiopia, wherein the authors accounted that people having better income were significantly associated with increased ITN utilizations (AOR = 1.83; 95 % CI 1.05−3.20) as higher socioeconomic status people can afford to buy and replace their damaged bed nets [32].

The study results also indicated that lower socioeconomic status, not having sufficient knowledge about malaria and LLINs, not having formal education and not having a stable job were some of the barriers to LLINs ownership and proper utilization of bed nets.

As regards to the overall knowledge about malaria among mothers in intervention villages, there was an improvement in knowledge from 3.8 to 34.2 % (p < 0.05) (η^2^ = 0.64). Similar results were reported by Adebayo et al. who found improvement of general knowledge about prevention and control of malaria [34], and by Nwana in 2011 who found that 56.12 % of the respondents had a good knowledge of malaria prevention and control after intervention [25]. Contrasting results were reported by from Uganda by Mwanje in 2013 who found poor knowledge about malaria prevention and control (20 %) among the study subjects [36].

With respect to overall attitude about malaria among mothers in intervention villages 75.2 % of mothers had a positive attitude towards malaria, which significantly increased to 99.3 % after the intervention (p < 0.05), (OR = 2.25). About the overall score of practice of malaria prevention in intervention villages, 0.8 % of mothers of under-five children, had good preventive practices against malaria that significantly increased to 33.6 % after the intervention (p < 0.05) (η^2^ = 0.79). Similar results were reported by Ibrahim et al. who found that high knowledge was the only predictor of high mean practice score [37]. A higher result was obtained by Envuladu et al. who found that the health education intervention increased practice of sleeping under the nets considerably 89 % [23].

## Conclusion

Following 10 months of COMBI activities related to malaria prevention and control, a significant increase was seen in mothers’ knowledge regarding malaria transmission, prevention and control. Also mothers developed more positive attitudes regarding prevention and control of malaria. In addition, increased use of LLINs was observed following intervention. No such changes were reported among mothers who were living in comparison villages. Based on the findings of this study, it is recommended that there is a need to concentrate on COMBI strategies to enhance utilization of LLINs through all available channels.

## Additional file

**Additional file 1.** Questionnaire.

## Abbreviations

COMBI: communication for behavioural impact
ITNs: insecticide-treated bed nets
PPM: personal protective measures
OR: odds ratio
WHO: World Health Organization
NMCP: National Malaria control programme
GFATM: global fund to fight tuberculosis and malaria
LLINs: long-lasting insecticidal nets
NGOs: non-governmental organizations
KAP: knowledge, attitude and practice
FGD: focus group discussion
SD: standard deviations
RDT: rapid diagnostic test
