# Supplementary material for: Communication for behavioural impact in enhancing utilization of insecticide-treated bed nets among mothers of under-five children in rural North Sudan: an experimental study
Source: Malar J. 2016 Oct 18;15:509. doi: 10.1186/s12936-016-1551-8 (PMC5070147; doi:10.1186/s12936-016-1551-8)
Supplement: Supplementary file 1 — Additional file 1. Questionnaire. [file 12936_2016_1551_MOESM1_ESM.doc]

**Questionnaire**

**First: Personal information:**

1. Gender:

-Male - Female

1. Age per years: ……………………
2. House hold occupation:

- Employee - Merchant - Farmer -Business man
- Others

1. Average of monthly income: …………………………..
2. What is family number: ………………………….
3. Number of children < 5 years: ………………………
4. Education level of household:

- Informal education - Basic/primary education
- Intermediate - Secondary
- University and post

1. Type of housing:

- Cement -Local materials - Others define: …
- **Second: information’s related with KAPs of mothers towards LLINs:**

9- Have you heard about malaria?

- Yes - No

`10- What is the vector of malaria parasite?

- Mosquitoes - Fly - Cockroach Others

11-how mosquito transmits malaria?

- By biting -By contact - I don't know
- Other (define): ………………………….

12- What are the signs and symptoms of malaria?

- Fever - Joints pains
- Vomiting and diarrhea - All above

13- Do you think malaria is serious disease?

- Yes - No

14- What is the treatment of malaria?

- Medicine (Drugs) -Traditional drugs
- Medicine and traditional drugs -I don't know

15- Do you think malaria is a preventable disease?

- Yes - No

16- If the answer in Q15 (yes). What are the methods of malaria prevention?

- Using insecticides -Using biological control
- Using environmental control - using repellants
- using personal protection measures - all above

17- If the answer in Q16- personal protection-define what are the personal protection measures used?

- ITNs - Repellants -All mentioned - Other (define)…

18- Have ITNs types?

- Yes - No

19- If the answer in Q18 (yes). What are the types of ITNs?

- Not- impregnated - Impregnated
- All mentioned -Other (define)… ……

20-The answers in Q19 (impregnated) have the impregnation types?

- Yes - No

21- If the answers in Q20 (yes) what are the types of impregnation?

- Long-lasting -ITNs -All mentioned

22-does ITNs differ from not- impregnated?

- Yes - No - I don't know

23-If the answers in Q22 (yes) what is the difference between the impregnated and non impregnated nets?

- Impregnation increase the efficacy of the ITNs
- ITNs have high quality
- Others (define)………………..

24- Can ITNs help in prevention of malaria?

- Yes - No - I don't know

25-How ITNs can prevent man against malaria?

- Making barrier between man and mosquito
- Escape mosquito away from man
- Killing mosquito - All mentioned

26- Do you have ITNs in your house?

- Yes - No

27. If yes, then, (you have ITNs in your house), how many ITNs do you have?

- With at least one net - Two net - Three and more net

28- If the answer to Q26 is (yes), then, do you use ITNs every day?

- Yes - No

29-If the answer to Q28 is (No).why you don’t use it?

- Difficulties in preparing and collecting INTs every days
- Difficulties in collecting INTs every days
- Climatic change - Harmful to the health
- Uncomfortable - No mosquitoes -Not having enough nets

30- Do you think that more large holes ITNs protect man from malaria?

- Yes - No

31- Do you think that ITNs can be an effective means against mosquito biting?

- Yes - No

32. Do you know the proper way of using ITNs?

- Yes - No - To some extent

33-If the answer to Q32 (yes or to some extent).what is the correct from the following options?

- Put it every well under the mattress
- Put it partially under the mattres
- I don't put it under the mattress
- Using sticks in preparing ITNs

34- Inside the house, what is the site where you use ITNs?

- Indoor - Outdoor - Both in and out door

35- In Emergency case, when you leave ITNs while you sleep .what is the situation of ITNs to be?

- Put it under the mattress well - Leave it free Let some holes

36-when you leave it without folding it under the mattress .do you expel mosquito inside ITNs away before sleeping

- Yes - No

37-In which season do you use bed nets in or out door?

- Autumn - winter - summer -all seasons

38- In which season of the year you use ITNs?

- all the year - Autumn - winter - summer

39-At what time of the day do you prepare ITNs?

- At the night - Before sunset -After sunset -During the day

40- To whom do you always give priority for sleeping under ITNs?

- Children <5 years - Pregnant women
- Elders - All family member

41- Do you face any difficulties in preparing and collecting ITNs?

- Yes - No

42-What are these difficulties?

………………………………………………………………………………………………………………………………………

43-1-If you have ITNs for each member of your family. Do you encourage them to use it?

- Yes - No

44-If answer to Q43 is (no).-why not encourage using?

- ITNs sticks are not available for everybody in my family
- Small area of my house
- Difficulty in collecting ITNs ,so it may late pupils going to school
- Harmful to the health
- Uncomfortable

45-How do you save ITNs during the day?

- At the house yard under the sun light -Inside the room
- Inside the food store -At the kitchen or toilet

46-How do you keep ITNs clean?

- By washing
- Avoidance to be contacted with the dusts
- All above
- Leave it without using

47-When you keep ITNs by washing. Define the period of times that you wash it?

- Once every month - Once every 6 month -Four times every year

48- After washing the ITNs. How do you dry it?

- Under the sun - Under the shadow - By using electric drier

49- Was anyone of your family was infected by malaria during last two weeks?

- Yes - No - I don't know

50- If the answer is yes, who was infected by malaria?

- Male/female <5child - Pregnant lady
- Elder - >5 yr child
- Others …………..

51- If yes to Q49, what could be the reason of malaria infection?

- They have not used ITNs ever - Low immunity
- Not used ITNs continuously - More contact with mosquito

52-What are your views about ITNs as a mean of preventive measures against malaria?

- Positively -Negative - Some extent

53-What are your information sources about malaria?

- Health promotion team
- Audio-visual media (TV)
- Audio media (Radio)
- Newspaper
- Other means distributed by ministry of health
- School students
- Community leaders

54- Do you think that the general public can play any role in preventing malaria?

- Yes - No - I don't know

55-which roles general public can act in prevention of malaria?

- Participating in various activities of malaria control
- Prevent indoor breeding of mosquito
- Early treatment of malaria cases among family members
- Prevent outdoor breeding of mosquito
- Using multi-personal protection measures as ITNs
- Participating in health education campaigns

**Third : Opinion of mothers / household about the visit of health team to home (respondents from intervention villages)**

56- Have you been visited by a member of health promotion team?

- Yes - No

57- If answer yes in Q56, identify the frequency of this visit?

- Continually - Intermitted -We haven’t visited by anybody

58- Health promotion team checking the ITNs during their visits?

- Yes - No

59- Instructions given to mothers by health promotion team during their visits?

- The importance of using ITNs - how to utilize ITNs
- how to keep and use ITNs

60- Lifestyle changes due to the frequent visits of health promotion team

- Using ITNs continuously
- To participate in malaria control voluntarily
- Learnt that malaria is preventable disease
- No change
